# Integrated pharmaceutical care model by unit-based clinical pharmacists: Implementation and clinical impact

**DOI:** 10.1016/j.rcsop.2025.100700

**Published:** 2025-12-18

**Authors:** Kezhen Feng, Xinyan Han, Nan Lv, Chaogang Xiong, Yajing Li, Bo Yang, Jingjing Yi, Tao Zhang

**Affiliations:** a Key Laboratory of Shaanxi Province for Craniofacial Precision Medicine Research, College of Stomatology, Xi'an Jiaotong University, Xi'an, Shaanxi, China; b Clinical Research Center of Shaanxi Province for Dental and Maxillofacial Diseases, College of Stomatology, Xi'an Jiaotong University, Xi'an, Shaanxi, China; c Department of Pharmacy, College of Stomatology, Xi'an Jiaotong University, Xi'an, Shaanxi, China; d Department of Pharmacy, Shaanxi Second Provincial People's Hospital, Xi'an, Shaanxi, China; e Department of Pharmacy, Xi'an Hospital of Traditional Chinese Medicine, Xi'an, Shaanxi, China; f Department of Pharmacy, Xi'an Chest Hospital, Xi'an, Shaanxi, China; g Department of Epidemiology and Health Statistics, School of Public Health, Fudan University, Shanghai, China; h Key Laboratory of Public Health Safety, Ministry of Education, Shanghai, China; i Department of Neurology, The Third Affiliated Hospital of Xi'an Medical University, Xi'an, China

**Keywords:** Respiratory department, Clinical pharmacist, Pharmaceutical care model, Unit-based pharmacists

## Abstract

**Background:**

In response to China's National Health Commission mandates promoting unit-based clinical pharmacists, healthcare institutions face severe workforce shortages, creating a critical policy-resource disparity.

**Objective:**

This study aimed to implement and evaluate a hybrid unit-based clinical pharmacist model in respiratory wards to address this gap.

**Methods:**

A structured workflow was implemented, integrating morning clinical activities (joint physician-pharmacist rounds, medication reconciliation, and real-time interventions) with afternoon analytical tasks (medication order surveillance). The model was evaluated quantitatively from 2021 to 2024.

**Results:**

Post-implementation, antimicrobial use density dropped from 114.43 to 103.82 DDDs/100 patient-days (a 9.3 % reduction), dual antimicrobial therapy fell from 29.89 % to 11.34 % (a 62.1 % reduction), and pharmacist-patient interactions rose 3.3-fold. Medication safety was enhanced, with adverse drug reaction reports growing from 34 to 61 (a 79.4 % increase). Seven representative cases illustrated the resolution of critical drug therapy issues, demonstrating the framework's capacity to augment stewardship and safety despite staffing constraints.

**Conclusions:**

The hybrid model provides a scalable framework for hospitals addressing the clinical pharmacy staffing gap in China. By strategically allocating limited pharmacist resources, it enhances antimicrobial stewardship and medication safety while complying with national reforms.

## 1 Background

A pivotal shift in pharmacy practice, triggered by severe drug-related injuries in the mid-20th century, moved the profession from a drug-centered to a patient-centered paradigm.^1^^,^^2^ This paradigm shift established the role of the clinical pharmacist, who utilizes pharmacological expertise to optimize medication regimens through therapeutic drug monitoring, medication counseling, and evidence-based decision support in multidisciplinary rounds.^3^

Empirical evidence demonstrates that clinical pharmacist-led medication history acquisition at hospital admission mitigates medication errors by 51 %, while their participation in interdisciplinary ward rounds reduces errors by 29 % and decreases adverse drug reaction (ADR) incidence, directly contributing to reduced hospital length of stay and mortality.^4^^,^^5^ Globally, innovative roles such as United States (U.S.) medication-administration pharmacists—managing 99 % of inpatient drug administration—exemplify this expanded scope.^6^ However, even in high-resource settings, workflow inefficiencies persist, with pharmacists facing ∼34 daily interruptions despite spending 80 % of their time on clinical activities.^7^^,^^8^

In China, a critical pharmacist shortage (0.35/1000 population vs. 0.78 in U.S.) severely impedes such advancements.^9^ This deficit coincides with rising clinical challenges: newly recruited physicians often lack antimicrobial stewardship expertise amid escalating national antibiotic resistance rates.^10^ Although recent National Health Commission (NHC) policies mandate clinical pharmacy integration,^11^ existing models face irreconcilable trade-offs in resource-limited settings, lacking either real-time input, feasibility, or task-prioritization protocols.^9^^,^^12^^,^^13^

To bridge this policy-resource gap, a hybrid unit-based pharmacist model was pioneered in respiratory wards. This framework integrates proactive clinical services with essential pharmaceutical tasks within existing staffing constraints, strategically allocating pharmacist time to enable 24/7 coverage. This study evaluates the model's clinical impact, antimicrobial stewardship efficacy, and operational feasibility, providing a scalable template for low-resource health systems globally.

## 2 Methods

This retrospective, mixed-methods study (January 2021–December 2024) evaluated a hybrid unit-based clinical pharmacist model using: (a) Quantitative analysis: antimicrobial use density, combination therapy rates, antibiotic combination rate (%), rate of specimen submission prior to restricted antimicrobial prescribing (%), rate of appropriate antimicrobial prescribing in inpatients (%), number of consultations and monitoring for inpatients, proportion of inpatients receiving pharmacist consultations/monitoring (%), number of reported ADRs, ADRs reporting rate (%). (b) Qualitative case review: 7 representative cases selected from 827 pharmacist interventions.

### 2.1 Operational framework

**Fig. 1:**
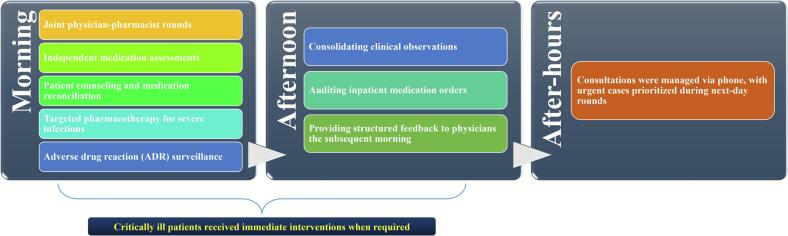
Workflow of unit-based clinical pharmacists in respiratory ward.

Morning clinical duties for the unit-based clinical pharmacist included joint rounds, medication reconciliation, and targeted pharmacotherapy management (left-side box in Fig. 1). Afternoon analytical tasks encompass audit of medication orders and synthesis of structured feedback (middle-box in Fig. 1). Critical cases prompted immediate interventions. Secure messaging provided 24/7 consultation support, with urgent issues escalated to next-day joint rounds (right-side box in Fig. 1).

### 2.2 Training protocol

Thirty-six evidence-based trainings during clinical handovers covered topics including antimicrobial resistance mechanisms, pharmacokinetics/pharmacodynamics (PK/PD)-guided dosing optimization, pharmacotherapy for special populations, and pattern analysis of ADRs, enhancing departmental antimicrobial stewardship capabilities.

The clinical pharmacists implementing this model were all qualified professionals who had completed the national standardized one-year clinical pharmacy residency training and obtained the corresponding certification. Before undertaking independent clinical responsibilities within the hybrid model, each pharmacist was required to complete a period of supervised clinical practice under a senior clinical pharmacist. This mandatory supervision period lasted at least one year, with the exact duration contingent upon an individualized competency assessment. Competencies assessed included comprehensive medication review, evidence-based intervention, pharmacotherapy monitoring, and the management of complex drug therapy issues. Independent practice was only permitted upon satisfactory evaluation and approval by the supervising senior pharmacist.

### 2.3 Integrated pharmacotherapy rounds: collaborative and pharmacist-led models

Joint physician-pharmacist rounds integrate medical and pharmaceutical perspectives, complementing routine medical assessments. Pharmacists specialized in obtaining comprehensive medication histories and allergy profiles, ensuring balanced consideration of efficacy and safety during therapeutic optimization. During pharmacist-led rounds, treatment response and ADRs were independently monitored, while concurrently delivering medication education and reconciliation. For severe infections, daily joint rounds were initiated at admission to establish evidence-based regimens, followed by scheduled reassessments every 72 h, with as-needed adjustments made in case of clinical deterioration. Pharmacists conducted supplemental medication reviews between joint sessions. Non-severe cases receive 1–2 pharmacist-led assessments during hospitalization, with escalation to joint rounds if suboptimal response (typically occurring between Days 3–5 post-initiation) or ADRs emerged. Pharmacists monitored such cases until resolution of therapeutic concerns.

Case 1 A 67-year-old male with acute exacerbation of chronic obstructive pulmonary disease (COPD) and a documented β-lactam allergy, received ciprofloxacin 0.4 g, q12 h. On day 5, he developed fever (38 °C) with unimproved dyspnea. During joint rounds, the pharmacist identified a history of a prior cefazolin-induced rash (non-systemic). Per side-chain theory, cefoperazone-sulbactam 3 g, q8 h was initiated given low cross-reactivity risk.^14^ Subsequent pharmacist-led rounds confirmed resolution of fever within 24 h and progressive symptom improvement. The patient was discharged on day 6 without adverse events.

Case 2 A 42-year-old female with community-acquired pneumonia (CAP) presented with a cough that was initially described as productive but was predominantly dry. Despite a weakly positive Mycoplasma pneumoniae DNA test and prior treatment failure with azithromycin, levofloxacin 0.5 g daily was initiated. On day 4, sputum production improved but nocturnal cough exacerbation persisted. During joint rounds, the pharmacist identified household exposure to two pediatric Mycoplasma infections. Suspecting azithromycin-resistant Mycoplasma pneumoniae, doxycycline 0.1 g, q12 h was added.^15^ Cough frequency decreased within 24 h, resolving significantly by day 3. The patient was discharged on day 5 with instructions for extended doxycycline therapy (5–7 days).

### 2.4 Medication surveillance

Critically ill patients with complex infections frequently exhibited comorbidities including advanced age, organ dysfunction, and polypharmacy. These cases demand comprehensive pharmacotherapy surveillance spanning admission to discharge, necessitating careful initial regimen design and ongoing therapeutic monitoring.

Case 3 An 88-year-old male (60 kg; baseline serum creatinine [SCr] 161 μmol/L) with recurrent severe pneumonia and fungal urinary tract infection presented with sustained fever (38.3–38.5 °C), altered consciousness, tenacious yellow sputum, and purulent urine. Initial joint rounds led to cefoperazone-sulbactam 1.5 g, q6 h plus fluconazole (loading dose 0.4 g, maintenance 0.2 g daily) for suspected polymicrobial infection. On day 3, acute cardiopulmonary decompensation requiring mechanical ventilation emerged, with worsening renal function (SCr 232 μmol/L) and elevated inflammatory markers. Pharmacist-guided meropenem adjustment (1 g loading → 0.5 g q6 h as 2 h-infusions) followed PK/PD principles. By day 5, urine culture confirmed Candida krusei (fluconazole-intermediate) while blood smear revealed fungal elements, suggesting hematogenous dissemination. Empirical micafungin (50 mg IV daily) and amphotericin B bladder irrigation (25 mg/L in sterile water) were initiated pending species identification.^16^ Defervescence occurred within 72 h. Bronchoalveolar lavage fluid culture subsequently identified *C*. tropicalis (micafungin-sensitive/fluconazole-resistant), validating the regimen. Post-extubation (day 7), symptoms resolved coinciding with normalized β-D-glucan (227.9 → 10.6 pg/mL). The patient completed 14 additional treatment days without renal deterioration before day-26 discharge.

Case 4 A 34-year-old obese female (85 kg) with CAP presented with diabetic ketoacidosis and poorly controlled type 2 diabetes. She had persistent high-grade fever (39 °C) for 3 days pre-admission with violent dry cough. After self-administering 1.2 g moxifloxacin within 24 h, she developed tachycardia (140 bpm). During emergent joint rounds, pharmacists confirmed the ingestion had occurred >5 h earlier, precluding gastric decontamination. Cardiac monitoring, fluid resuscitation, and β-blockers were initiated. Despite negative Mycoplasma PCR, ongoing violent cough and nationwide Mycoplasma prevalence prompted initiation of amoxicillin-clavulanate 1.2 g, q6 h IV and doxycycline 0.1 g, q12 h, PO. Temperature normalized to 36.5–36.7 °C by day 3 with cough improvement. On day 4, transient fever spiked to 38.5 °C, progressing to sustained pyrexia (>38 °C) by day 5. Repeat joint rounds considered Pseudomonas aeruginosa infection due to uncontrolled diabetes,^17^ leading to piperacillin-tazobactam 4.5 g, q6 h. Afebrile status (<37.3 °C) was achieved within 24 h of regimen change, with complete symptom resolution by day 6. At discharge, pharmacists provided comprehensive counseling on glycemic control and medication safety.

### 2.5 Medication reconciliation

Pharmacist-led medication reconciliation demonstrably mitigates care-transition risks and reduces medication errors—particularly for untreated indications and unmanaged adverse drug events where pharmacist-led medication reconciliation outperforms physician-directed processes.^13^ In China, however, this remains in pilot implementation with 9.6 % reduction in medication discrepancies, though significant, falls below the 11–52 % range documented in multinational studies (U.S., Canada, Netherlands, Colombia, Ireland), highlighting context-specific implementation challenges.^18^ Elderly patients with multimorbidity and polypharmacy face elevated risks of drug interactions and adverse reactions.^4^^,^^18^ Systematic reconciliation optimized essential medications while maintaining efficacy.

Case 5 A 72-year-old male with acute COPD exacerbation and stable angina developed tachycardia (HR 100 bpm) 7 h post-admission, resolving with oxygen and positioning. Pharmacist-led rounds identified therapeutic duplication: home medications (aminophylline tablets, compound ephedrine-theophylline tablets, salbutamol tablets, prednisone) overlapped with inpatient orders (doxofylline injection, salbutamol aerosol, methylprednisolone). Concomitant theophylline products likely induced tachycardia via supratherapeutic concentrations.^19^ The pharmacist recommended: (i) Discontinuing prednisone, aminophylline, and compound ephedrine-theophylline during hospitalization, transitioning to doxofylline tablets post-discharge; (ii) Substituting oral salbutamol with an inhaled formulation (faster onset, lower systemic exposure). No further palpitations occurred. Discharge counseling emphasized avoiding self-adjustments and consulting professionals for regimen changes.

### 2.6 Medication education

For inhaled therapies (corticosteroid-containing aerosols/powders), pharmacists emphasized adherence and mandatory post-inhalation rinsing.^20^ For extended antimicrobial courses (e.g., lung abscess/pulmonary mycosis requiring 6–8 weeks), patients received intravenous therapy for ∼2 weeks before transitioning to oral agents. Pre-discharge counseling targeted patients considering premature treatment discontinuation upon symptomatic improvement, emphasizing completion of the full therapeutic course, dosage regimens and administration techniques, potential adverse effects and precautions.

### 2.7 Pharmacist-led medication order review

Pharmacists performed randomized audits of respiratory ward medication orders, identifying inappropriate prescriptions including undocumented infections, incorrect dosing, and unjustified antimicrobial combinations, and inadequate progress notes. Interventions involved direct physician communication with evidence-based recommendations. Recurrent issues were systematically categorized and presented during morning handovers.

Case 6 Initial audits revealed absent antimicrobial justification in medical records, preventing rationality assessment. Pharmacists developed a disease-specific documentation template covering CAP, COPD exacerbations, and bronchiectasis infections, requiring documentation of rationale for initial antimicrobial selection, therapy modifications during hospitalization, combination regimens, adverse reaction-related adjustments, ongoing pharmacist-physician collaboration enhanced documentation completeness.

Case 7 Between February–April 2022, five cases of unjustified meropenem prescribing were identified despite pharmacist objections. Data analysis showed meropenem consumption ranked top-quartile hospital-wide during Q1 2022. Pharmacists developed targeted prescribing recommendations, disseminated during morning educational sessions. Enhanced surveillance reduced meropenem utilization and inappropriate prescriptions.

## 3 Results

**Fig. 2:**
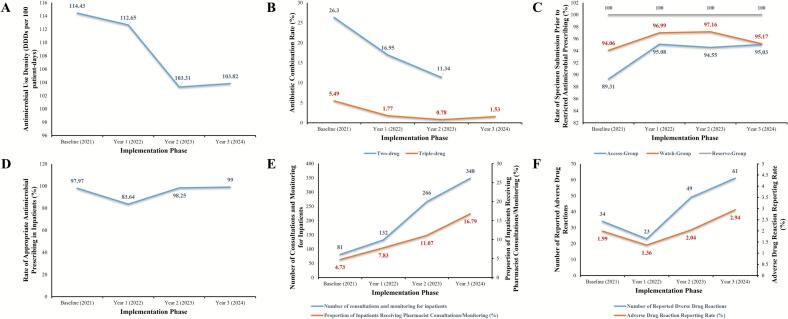
Comparative analysis of key metrics before and after implementation of the unit-based clinical pharmacist model in respiratory wards. (A) antimicrobial use density (defined daily dose per 100 patient-days); (B) antibiotic combination therapy rate (%); (C) Specimen Submission Rate Before Restricted Antimicrobial Use (%); (D) rate of appropriate antimicrobial prescribing in inpatients (%); (E) number and proportion of inpatients receiving pharmacist consultations/monitoring (%); (F) number and proportion of reported adverse drug reactions.

Post-implementation outcomes included a 3.3-fold increase in pharmacist-patient interactions (2021–2024), sustained reductions in antimicrobial use density (from 114.43 to 103.93 DDDs per 100 patient-days), reductions in combination therapy utilization (dual therapy: 29.89 % to 11.34 %; triple therapy: 5.49 % to 1.53 %), improved rates of specimen submission prior to prescribing acccess-group antimicrobials (from 89.31 % to 95.03 %), a transient decline followed by recovery in the rate of appropriate antimicrobial prescribing (from 97.97 % to 99.00 %), a resurgence in ADR reporting following an initial decline (from 34 to 61 cases reported). The department received the hospital's 2023 Rational Medication Practice Award, reflecting multidisciplinary recognition of the model's impact (Fig. 2).

## 4 Discussion

In recent decades, evolving evidence has driven a paradigm shift in hospital pharmacy practice—from medication dispensing to patient-centered services^21^ A synthesis of 18 studies (published in BMJ, JAMA, The Lancet, and NEJM) demonstrates the critical role of clinical pharmacists in enhancing therapeutic outcomes and patient quality of life.^22^ Evidence for this role transformation continues to solidify. Beyond scoping reviews of top-tier journals, a recent systematic review has further quantified its impact, indicating that systematic clinical pharmacy services can reduce medication discrepancies by approximately 30 % and tangibly improve healthcare quality and safety by reducing unnecessary readmissions.^23^ Furthermore, forward-looking research is beginning to explore the role of pharmacists in more complex clinical scenarios, such as identifying and preventing “prescription cascades” (where new prescriptions are initiated to treat adverse drug reactions).^24^ This continuously expands the value boundaries of pharmaceutical care and demands greater depth and breadth from its service delivery models.

China has implemented frameworks for reimbursing pharmaceutical care services to operationalize this transition, with recent NHC policies incentivizing healthcare institutions to expand clinical pharmacy services while ensuring equitable compensation and professional recognition—a structural breakthrough validating the clinical role of pharmacists.^25^ However, the density of pharmacists in China (0.35 per 1000 population in 2020) remains significantly lower than the U.S. benchmark (0.782 per 1000). Pharmacists comprised only 4.65 % of China's 10.7 million healthcare professionals in 2020. Despite an average annual growth rate of 3.15 % from 2016 to 2020, this expansion rate lagged behind that of the overall healthcare workforce. Consequently, the proportional representation of pharmacists declined persistently from 5.20 % to 4.65 %, exacerbating disparities in the provision of clinical pharmacy services.^10^

The transition from fragmented services toward integrated models represents a clear trend in international pharmacy practice. Recent scholarship indicates that advanced pharmaceutical care has evolved from isolated medication reconciliation to a continuous service framework spanning the entire patient hospitalization cycle, with deep collaboration within the healthcare team at its core.^26^ This global trend resonates with the ongoing value reconstruction within hospital pharmacy departments in China—shifting from a drug-centered “profit center” to building a “patient-centered service value chain,” where the current core challenges remain singular service models and insufficient interprofessional collaboration.^27^ Consequently, exploring an efficient model that can systematically integrate core pharmaceutical services and enable real-time collaboration with clinical teams, despite the rigid constraint of pharmacist shortages, has become a pivotal task for advancing clinical pharmacy practice in China. The hybrid workflow model developed in this study constitutes a localized response to this urgent need.

While patient-centered clinical pharmacy services manifest through three documented models.^7^ Model 1 (retrospective order verification without ward rounds) delivers fragmented impact due to the lack of real-time clinical input. Conversely, Model 2 (dedicated patient care involving daily rounds) is operationally unfeasible for over 95 % of Chinese hospitals, including tertiary centers, due to workforce shortages (with fewer than 0.4 pharmacists per bed available).^10^ Although Model 3 (decentralized satellite pharmacy with mixed dispensing/prospective review) offers higher scalability, its effectiveness depends critically on strategic task prioritization, for which evidence-based protocols are currently lacking.

This model was pioneered prior to the commencement of formal national pilot programs. The hybrid operational framework, while fully compliant with regulatory standards encompassing medication order review, joint ward rounds, pharmacotherapy surveillance, medication reconciliation, and patient education, was specifically designed to address institutional resource constraints.^28^ Pharmacists engaged in direct clinical activities within the wards during morning hours, undertook pharmaceutical duties (including dispensary operations and therapeutic data analysis) in the afternoons, and provided 24/7 emergency coverage, requiring immediate attendance for critical cases. Continuous digital consultation via phone enabled real-time medication adjustments during off-hours, particularly for intensive care patients experiencing acute deterioration. This deliberate partitioning of tasks and time is designed not only to integrate clinical and dispensing functions but also to enhance overall workflow efficiency. Studies have shown that optimizing pharmacy workflows through task restructuring and digital tools can significantly reduce time spent on logistical activities, thereby reallocating valuable human resources to direct patient care.^29^

The core operations of this hybrid model, such as structured medication reconciliation and regular participation in ward rounds, share conceptual ground with internationally validated successful paradigms. For instance, the Lund Integrated Medicines Management model, originating in Sweden and adapted in Japan, has demonstrated significant improvements in patient safety and continuity of care through systematic pharmacist-led medication assessment and inter-team communication, affirming the effectiveness of such integrated service frameworks.^30^ The novel hybrid unit-based clinical pharmacist model fundamentally reoriented practice from dispensary-based operations to active engagement within the wards, transforming passive retrospective review into proactive real-time intervention. This paradigm shift enabled the preemptive identification and correction of medication errors during critical treatment phases, establishing pharmacists as essential gatekeepers of pharmacotherapy safety.^31^ By integrating directly with clinical teams at the point of care, pharmacists collaborated dynamically with physicians and nurses to resolve complex therapeutic challenges more efficiently.

The deep clinical integration and multidisciplinary trust fostered by this model align with positive feedback from international practice. Studies have shown that integrating clinical pharmacists into specialty clinics in the United States not only improved clinical continuity by 23 % but also significantly increased the acceptance of pharmacists as decision-making partners by the physician-nurse team.^32^ This supports the notion that when pharmaceutical care is deeply embedded in the clinical workflow, pharmacists can transcend traditional reviewing roles to become dynamic collaborative forces in solving complex therapeutic challenges and enhancing overall care quality.

Several significant limitations of this model and the current clinical pharmacy landscape in China warrant careful consideration and future resolution. First, the hybrid model's intensive clinical integration came at the cost of increased pharmacist workload and dependence on extended hours, a direct reflection of the severe underlying constraint of pharmacist understaffing. This limitation is symptomatic of broader systemic challenges: the nascent stage of clinical pharmacy practice in China, variable levels of pharmacist expertise, and the profession's relatively low institutional status and compensation collectively constrain the scope, sustainability, and motivation for deep clinical engagement. Consequently, even with extraordinary effort, the model could not yet provide comprehensive pharmacotherapy coverage for all patients in the ward. Second, operational constraints were noted. The absence of intelligent prescribing systems meant some errors were missed during manual order reviews, highlighting the need for AI-powered screening software.^33^ Concurrently, the lack of therapeutic drug monitoring platforms for agents like voriconazole and vancomycin prevented personalized dosing in critically ill patients, posing risks of subtherapeutic efficacy or toxicity. To address these constraints and ensure the model's scalability, future iterations should integrate technological enablers such as automated dispensing systems and AI-powered prescribing support. Evidence indicates that pharmacy automation and digital workflow management are pivotal strategies for mitigating human resource shortages, reducing error rates, and creating the capacity necessary for expanding clinical services.^34^ Therefore, combining the “clinical expertise of pharmacists” in this model with the “efficiency of artificial intelligence” to establish a new “human-machine collaborative” mode for prescription review and monitoring represents a critical direction for overcoming current manpower bottlenecks and achieving a dual leap in both quality and efficiency.

However, these limitations precisely delineate the critical path forward. The demonstrated success of this model—achieving deep clinical integration, building substantial multidisciplinary trust, and improving key outcomes—provides a powerful evidence base for change. It argues compellingly for parallel investments in the clinical pharmacy workforce: strategic expansion of positions, enhanced and standardized training to elevate expertise, and improved professional recognition. The ultimate goal should be the normalization and synchronization of clinical pharmacy services, moving from an over-reliance on individual dedication to a sustainably staffed system capable of providing high-quality, 24/7 pharmaceutical care. Future work must focus on formal workload assessments, cost-benefit analyses, and the development of supportive technologies to ensure the scalable and sustainable implementation of such integrated models.

## 5 Conclusion

Overall, this study presents an innovative unit-based clinical pharmacist model delivering resource-efficient bedside care, demonstrating dual efficacy in optimizing patient outcomes; its operational flexibility provides a scalable framework for embedding pharmacists in multidisciplinary teams—reconciling therapeutic excellence with sustainable resource utilization.

## CRediT authorship contribution statement

**Kezhen Feng:** Writing – original draft, Formal analysis, Data curation, Conceptualization. **Xinyan Han:** Writing – review & editing, Writing – original draft, Supervision, Conceptualization. **Nan Lv:** Formal analysis, Data curation. **Chaogang Xiong:** Data curation. **Yajing Li:** Data curation. **Bo Yang:** Writing – original draft. **Jingjing Yi:** Writing – review & editing, Writing – original draft, Conceptualization. **Tao Zhang:** Writing – review & editing, Writing – original draft, Supervision, Conceptualization.

## Consent for publication

Not applicable.

## Ethical approval and consent to participate

The ethics has been approved by the Ethics Committee of the Shaanxi Second Provincial People's Hospital. Informed consent for the use of routinely collected data has been waived according to local requirements. All associated procedures were conducted in accordance with the approved guidelines.

## Declaration of competing interest

The authors declare no competing interests.

## Data Availability

The data used and analyzed during the current study are available from the corresponding author on reasonable request.

## Appendix A Supplementary data

Supplementary material: Supplementary Table S1. Comparative Analysis of Key Outcomes Before and After Implementation of the Hybrid Unit-based Clinical Pharmacist Model in Respiratory Wards Image 1
