# Supplementary material for: Integrated pharmaceutical care model by unit-based clinical pharmacists: Implementation and clinical impact
Source: Explor Res Clin Soc Pharm. 2025 Dec 18;21:100700. doi: 10.1016/j.rcsop.2025.100700 (PMC12811635; doi:10.1016/j.rcsop.2025.100700)
Supplement: Supplementary file 1 — Supplementary material: Supplementary Table S1. Comparative Analysis of Key Outcomes Before and After Implementation of the Hybrid Unit-based Clinical Pharmacist Model in Respiratory Wards [file mmc1.docx]

**Table S1. Comparative Analysis of Key Outcomes Before and After Implementation of the Hybrid Unit-based Clinical Pharmacist Model in Respiratory Wards**

| Outcomes | Implementation Phase | | | |
| --- | --- | --- | --- | --- |
|  | Baseline (2021) | Year 1 (2022) | Year 2 (2023) | Year 3 (2024) |
| Antimicrobial Use Density (DDDs per 100 patient-days) ^a^ | 114.43 | 112.65 | 103.31 | 103.82 |
| Antimicrobial Combination Therapy Rate (%) |  |  |  |  |
| Two-drug | 26.3 | 16.95 | 11.34 | 11.51 |
| Triple-drug | 1.77 | 0.78 | 1.53 | 1.26 |
| Specimen Submission Rate Before Restricted Antimicrobial Use (%) |  |  |  |  |
| Access Group | 95.08 | 94.55 | 95.03 | 93.95 |
| Watch Group | 96.99 | 97.16 | 95.17 | 97.72 |
| Reserve Group | 100 | 100 | 100 | 100 |
| Rate of Appropriate Antimicrobial Prescribing in Inpatients (%) | 97.97 | 83.64 | 98.25 | 99.00 |
| Number of discharges | 1711 | 1685 | 2402 | 2073 |
| Number of Consultations and Monitoring for Inpatients | 81 | 132 | 266 | 348 |
| Proportion of Inpatients Receiving Pharmacist Consultations/Monitoring (%) | 4.73 | 7.83 | 11.07 | 16.79 |
| Number of Reported Adverse Drug Reactions | 34 | 23 | 49 | 61 |
| Adverse Drug Reaction Reporting Rate (%) | 1.99 | 1.36 | 2.04 | 2.94 |

^a^ DDD: Defined Daily Dose. Antimicrobial use density is expressed in DDDs per 100 patient-days.
